# Collection Overview: Ten Years of Wonderful Open Access Science

**DOI:** 10.1371/journal.pbio.1001688

**Published:** 2013-10-22

**Authors:** Roland G. Roberts, Jane Alfred

**Affiliations:** *PLOS Biology*, Public Library of Science, Cambridge, United Kingdom

## Abstract

To mark our tenth Anniversary at *PLOS Biology*, we are launching a special, celebratory **Tenth Anniversary** ***PLOS Biology*** **Collection** which showcases 10 specially selected *PLOS Biology* research articles drawn from a decade of publishing excellent science. It also features newly commissioned articles, including thought-provoking pieces on the Open Access movement (past and present), on article-level metrics, and on the history of the Public Library of Science. Each research article highlighted in the collection is also accompanied by a PLOS Biologue blog post to extend the impact of these remarkable studies to the widest possible audience.

## Abstract 2

Announcing our Tenth Anniversary *PLOS Biology* Collection, Roli Roberts and Jane Alfred introduce the top 10 papers as selected by our Editorial Board Members and the Editorial team.

As we celebrate 10 years of *PLOS Biology*, 10 years of the Public Library of Science, and 10 years of strong advocacy and trail-blazing for the Open Access movement, we mustn't forget the real star of the show – the fantastic science that we've published.

It's hard to cast one's mind back 10 years and recall the scepticism with which open access publishing was initially received. A key concern at the time was that the model would be tainted with the stigma of “vanity publishing,” and that this model, in which the author pays to publish, is incompatible with integrity, editorial rigour, and scientific excellence. As also discussed in the accompanying editorial [1], the sheer quality of the science that has appeared in *PLOS Biology* has been vital for dispelling this myth.

Our tenth anniversary provides us with a great opportunity to celebrate all of the 1800 or so research articles published in *PLOS Biology* since our launch in 2003. Unable to showcase each one in turn, we turned to our Editorial Board to help us pick the top 10 research articles to feature in a special Tenth Anniversary *PLOS Biology* Collection (www.ploscollections.org/Biology10thAnniversary). During the month of October, we will also publish a PLOS Biologue blog post (http://blogs.plos.org/biologue/) for each of these selected research articles, trying to capture and convey what it is about them that the staff editors, the editorial board, and the authors feel is special.

By now, you're probably wondering which papers we selected. The selection is detailed in Box 1, with links to each article. If you haven't read these articles before, we urge you to read them now and to judge for yourself. As Editorial Board Member Steve O'Rahilly put it, “I think a common theme in many of the best *PLOS Biology* papers is that they are rich in data that is analysed very carefully and self-critically and presented without hype. However the conclusions are important for the biological community and their insights are likely to stand the test of time.”

As well as publishing research articles, *PLOS Biology* has a thriving Magazine section that has hosted scientific and policy debates, aired polemical and provocative views, celebrated scientific lives in obituaries, reviewed interesting books, and explored unsolved mysteries. One example of how this section has triggered productive community debate is Rosie Redfield's Perspective on how genetics should be taught to undergraduates [2]. Yet we don't seek just to provoke debate, but also to enlighten; take a moment to read Georgina Mace's editorial on the current issues and debates in the sustainability sciences [3]. We also try to break down barriers between fields [4] and to promote public engagement with science [5],[6].

We feel strongly that our role doesn't end with publishing the research article itself. Instead, we aim to unpackage the fascinating discoveries published in *PLOS Biology* by commissioning articles that explain the significance and impact of the research we publish to audiences of varying expertise. These companion articles range from Primers, which are written by experts who contextualise research articles for those in the field; to Synopses, which are written by science writers who digest an article for our wider readership of biologists; and finally, to PLOS Biologue blog posts, which distil research discoveries for a more general scientifically engaged public. We also use social media to bring these findings to the attention of a global online audience.

Of course, the continued success of *PLOS Biology* doesn't rest solely on the amazing research we've already published; it also hinges on the ground-breaking science we strive to publish in the future. Maintaining the high quality of the biology that we publish is of vital importance to us, not least because, as Editorial Board Member Robert Insall reflects, “What I like about *PLOS Biology* is that it avoids other journals' fixation on fashion and the biggest names. This means the papers *PLOS Biology* is publishing now will last longer and mean more in a generation's time.”

**Box 1. Research Articles Featured in the Tenth Anniversary *PLOS Biology* Collection**

Our Editorial Board Members helped us select 10 articles from the great science published during *PLOS Biology*'s first decade to feature in our Tenth Anniversary Collection. Please access these articles from the list below and from our Collection page. To read the PLOS Biologue blog posts that accompany them, please go to http://blogs.plos.org/biologue/ for more information.

Carmena J et al. (2003) Learning to Control a BrainMachine Interface for Reaching and Grasping by Primates

 Primer: Current Approaches to the Study of Movement Control

 Synopsis: Retraining the Brain to Recover Movement

Brennecke J et al. (2004) Principles of MicroRNA–Target Recognition

 Synopsis: Seeds of Destruction: Predicting How microRNAs Choose Their Target

Voight BF et al. (2005) A Map of Recent Positive Selection in the Human Genome

 Synopsis: Clues to Our Past: Mining the Human Genome for Signs of Recent Selection

Palmer C et al. (2007) Development of the Human Infant Intestinal Microbiota

 Synopsis: Microbes Colonize a Baby's Gut with Distinction

Levy S et al. (2007) The Diploid Genome Sequence of an Individual Human

 Synopsis: A New Human Genome Sequence Paves the Way for Individualized Genomics

Illingworth R et al. (2008) A Novel CpG Island Set Identifies Tissue-Specific Methylation at Developmental Gene Loci

Silva J et al. (2008) Promotion of Reprogramming to Ground State Pluripotency by Signal Inhibition

 Synopsis: A Shortcut to Immortality: Rapid Reprogramming with Tissue Cells

Coppé J-P et al. (2008) Senescence-Associated Secretory Phenotypes Reveal Cell-Nonautonomous Functions of Oncogenic RAS and the p53 Tumor Suppressor

Shu X et al. (2011) A Genetically Encoded Tag for Correlated Light and Electron Microscopy of Intact Cells, Tissues, and Organisms

Bonds MH et al. (2012) Disease Ecology, Biodiversity, and the Latitudinal Gradient in Income

 Synopsis: Which Came First: Burden of Infectious Disease or Poverty?
